# Origins of Knowledge: Insights from Precocial Species

**DOI:** 10.3389/fnbeh.2015.00338

**Published:** 2015-12-09

**Authors:** Elisabetta Versace, Giorgio Vallortigara

**Affiliations:** Animal Cognition and Neuroscience Laboratory, Center for Mind/Brain Sciences, University of TrentoRovereto, Italy

**Keywords:** origins of knowledge, precocial species, insects, avian species, *Drosophila*, *Gallus gallus*, domestic chicks

## Abstract

Behavioral responses are influenced by knowledge acquired during the lifetime of an individual and by predispositions transmitted across generations. Establishing the origin of knowledge and the role of the unlearned component is a challenging task, given that both learned and unlearned knowledge can orient perception, learning, and the encoding of environmental features since the first stages of life. Ethical and practical issues constrain the investigation of unlearned knowledge in altricial species, including human beings. On the contrary, precocial animals can be tested on a wide range of tasks and capabilities immediately after birth and in controlled rearing conditions. Insects and precocial avian species are very convenient models to dissect the knowledge systems that enable young individuals to cope with their environment in the absence of specific previous experience. We present the state of the art of research on the origins of knowledge that comes from different models and disciplines. Insects have been mainly used to investigate unlearned sensory preferences and prepared learning mechanisms. The relative simplicity of the neural system and fast life cycle of insects make them ideal models to investigate the neural circuitry and evolutionary dynamics of unlearned traits. Among avian species, chicks of the domestic fowl have been the focus of many studies, and showed to possess unlearned knowledge in the sensory, physical, spatial, numerical and social domains. Solid evidence shows the existence of unlearned knowledge in different domains in several species, from sensory and social preferences to the left-right representation of the mental number line. We show how non-mammalian models of cognition, and in particular precocial species, can shed light into the adaptive value and evolutionary history of unlearned knowledge.

## Introduction

Precocious knowledge can help naïve individuals in making correct predictions and deciding whether to approach or avoid an object and how to cope with a situation encountered for the first time.

Evidence of precocious knowledge has been documented in species with a short life span, when learning by trial and error could be too costly. For instance, in the absence of previous experience, fruit flies (*Drosophila melanogaster*) prefer to lay their eggs on fruit containing limonene, a behavior that helps them contrasting predation from parasitic wasps (Dweck et al., 2013). Examples of knowledge supported by little if any learning have been found also in species that live much longer than fruit flies, such as human beings and chicks of the domestic fowl (*Gallus gallus*), and in tasks that can involve less dramatic consequences in case of failure. Along with other species, young human beings know from the earliest stages of their life that a large object cannot be hidden behind a narrower occluder (Aguiar and Baillargeon, 1999, 2002; Chiandetti and Vallortigara, 2011b), that objects maintain their identity in spite of proximal changes on the retina (Wood and Wood, 2015), and continue to exist when occluded (Baillargeon et al., 1985; Regolin et al., 1995), that three dots are different from two (Rugani et al., 2009), 1/4 is different from 3/4 Rugani et al. (2014) and 4 is different from 12 (Izard et al., 2009), that an attractive face has two eyes and one mouth (Turati et al., 2002; Rosa-Salva et al., 2010), that cliffs are more dangerous than solid ground (Walk and Gibson, 1961).

The diverse examples of early knowledge can be conveniently grouped in two categories to help an interspecific comparison: (a) predispositions to attend or orient towards or away from particular stimuli (e.g., specific shapes (Fantz, 1957; Zanforlin and Vallortigara, 1985), colors (Hess and Gogel, 1954; Hess, 1956), and stimuli associated with social partners such as face-like stimuli (Johnson and Horn, 1988; Rosa-Salva et al., 2010), stimuli moving according to biological motion dynamics (Vallortigara et al., 2005; Vallortigara and Regolin, 2006; Simion et al., 2008) and (b) unconditioned assumptions/expectations about physical facts of the world that can in turn bias learning of certain stimuli or associations (e.g., constraints on learning (Garcia and Koelling, 1966; LoLordo, 1979) or preparedness for learning (Dunlap and Stephens, 2014) or help problem solving (e.g., object permanence (Regolin et al., 1995), physical reasoning about occluded objects (Chiandetti and Vallortigara, 2011b), use of geometrical information for spatial cognition and navigation (Tommasi et al., 2012).

In most of the cases, the origin of precocious knowledge and its evolutionary basis cannot be easily established. Knowledge available in the first stages of life can either be acquired through early specific stimulation—including embryonic experiences (Lecanuet and Schaal, 1996; Schaal et al., 2000; Graven and Browne, 2008; Harshaw and Lickliter, 2011)—or transmitted between generations, in the form of predispositions that orient perception, learning and other cognitive functions during the course of normal development. Precocial species, which are able to move and feed soon after birth and require little if any parental care, can have a great benefit from unlearned knowledge. Moreover, the life history of insect species who did not evolve parental care (Wong et al., 2013) gives them a privileged position for the investigation of unlearned knowledge. Insect species with no post-hatching parental care exhibit several unlearned abilities, such as phototaxis, thermotaxis and chemotaxis (e.g., Gong, 2012; Dubnau, 2014). In the aforementioned case of fruit flies and citrus preference, experimenters can control the environment to exclude a role of specific experience, try to identify the sensory-motor circuits that produce preferential choices, compare the ecology and oviposition choices of different species to investigate the adaptive value of a trait, or even run experimental evolution to investigate evolutionary responses at the behavioral and genetic level in a reasonable amount of time (Schlötterer et al., 2015; Versace, 2015). While most of studies involving insects focused on sensory preferences and preparedness for learning/constraints on learning (e.g., Giurfa et al., 1995; Dweck et al., 2013; Dunlap and Stephens, 2014), more recently other cognitive domains such as unlearned spatial cognition and navigation abilities (Lee and Vallortigara, 2015) and general learning abilities (Mery and Kawecki, 2002) have been investigated.

In the case of youngster of altricial species—including human beings, which are dependent on parental care for a long time, the options to investigate the origins of knowledge are much more limited. For centuries philosophers and scientists (reviewed in Hess, 1973; Spelke and Newport, 1998; Spelke, 1998) have been asking to which extent our perceptions and reactions depend on predisposed mechanisms disengaged from specific experience (nativist hypotheses) or on the specific experiences we have been exposed to (empiricist hypotheses). In the last years new techniques have been developed to measure precocious reactions and sensitivity to a variety of stimuli in infants (reviewed in Streri et al., 2013), that range from behavioral (e.g., habituation/dishabituation procedures (Aslin, 2007) to neuroimaging approaches (e.g., near infrared spectroscopy (Bartocci et al., 2000) and magnetoencephalography Cheour et al. (2004)). In spite of this, the range of capabilities that can be investigated soon after birth is still limited by the reduced range of motor actions and immaturity of perceptual abilities in infants.^1^

Moreover, for ethical and practical reasons primate babies cannot be raised in completely controlled rearing conditions or be deprived of important stimuli such as light or social contact until the moment of test, as it is done in isolation experiments.^2^ Wood and Wood (2015) have reviewed similar constraints in rodent species, for whom rearing in darkness prevents complete and normal maturation in the visual cortex, alters the development of GABAergic transmission, and cannot be raised from birth in controlled, lighted environments. In the light of the limitations that constrain research in altricial species, questions about the origin of unlearned knowledge might be more conveniently addressed using precocial species.

Avian precocial species (species that move around and feed on their own soon after hatching) such as young domestic chickens (*Gallus gallus*), goslings and ducklings have been used to investigate the existence and content of precocious knowledge in vertebrate species since the late nineteenth century (for a review, see Andrew, 1991). As for acoustic stimulation, Gottlieb (1968) showed that in ducks embryonic auditory experience with the own calls produces species-specific preferences for acoustical stimuli. In this species the acoustic system is tuned in such a way that the little experience gained by the embryo is sufficient to orient subsequent approaching responses to conspecific calls (Gottlieb, 1965). Similar results have been found in bobwhite quail chicks (Lickliter and Stoumbos, 1991). Prenatal learning of sounds has been observed in altricial species, including human beings (for a review, see Lecanuet and Schaal, 1996). As for visual stimuli, a wide range of experiments has identified preferences of naïve chicks to approach specific colors (Schaefer and Hess, 1951; Hess and Gogel, 1954), size (Schulman et al., 1970), shapes (Fantz, 1957; Zanforlin and Vallortigara, 1985), as well as configurations of static (e.g., face-like stimuli (Rosa-Salva et al., 2010) and dynamic stimuli (e.g., biological motion (Vallortigara et al., 2005), self-propulsion (Mascalzoni et al., 2010). The preferential orientation towards face-like stimuli has been attributed to the so-called CONSPEC system (Morton and Johnson, 1991). This mechanism specifies information on the general features of social partners and care-givers, as opposed to the so-called CONLERN system, devoted to learning the particular visual characteristics of specific individuals. Consistent with this hypothesis is the fact that precocial avian species which in the wild develop an attachment to the mother, share the preference for cues associated with social partners and care-givers with newborns of our species (e.g., Morton and Johnson, 1991; Simion et al., 2008). The co-occurrence of early preferences for face-like stimuli, biological motion and self-propelled objects suggests that social species might be endowed with a core knowledge system dedicated to social stimuli (Spelke and Kinzler, 2007; Vallortigara, 2012b).This suggestive hypothesis could be validated by investigating the differences in reactions to social stimuli between breeds and species that require a different degree of parental care soon after birth. Reptiles such as tortoises, which require no post-hatching parental care, are a good model system to understand the adaptive role and evolution of social core-knowledge mechanisms. While it has been showed that adult tortoises have the capability to follow the gaze of conspecifics (Wilkinson et al., 2010), little is known on their early social capabilities.

## Predispositions for Attractive and Aversive Stimuli

### Sensory Predispositions

Nativist theories have found support in the domain of sensory development (e.g., Walk et al., 1957; Walk and Gibson, 1961; Blakemore and Van Sluyters, 1975; Li and Liberles, 2015). The olfactory system of *Drosophila* is a good model for probing these theories. Compared to other sensory modalities, olfactory circuits require few connections to convert sensory inputs into behavioral responses [see CO_2_ avoidance (Suh et al., 2004)], and pheromone detection and courtship behavior (Dulac and Wagner, 2006; Ruta et al., 2010). Fruit flies exhibit consistent unlearned attraction and repulsion for food- and oviposition-related odors (Ruebenbauer et al., 2008; Semmelhack and Wang, 2009; Dweck et al., 2013; Min et al., 2013; Ronderos et al., 2014; Versace and Reisenberger, 2015) but these behavioral responses are not fixed. Peripheral (Krishnan et al., 1999, 2008; Root et al., 2011) as well as central circuits (Semmelhack and Wang, 2009) encode the valence of stimuli depending on odor-concentration—higher concentrations can recruit additional receptors, change central representations and modify the perceived valence (Semmelhack and Wang, 2009)—and the internal physiological state of the individual (e.g., circadian changes in olfactory sensory neurons have been observed (Krishnan et al., 1999, 2008); starved flies are differently responsive to odors compared to fed flies (Root et al., 2011). Hence in fruit flies attraction to food odors is state-dependent, both at the peripheral and central level.

The relative simplicity of the courtship behavior circuits in fruit flies makes the investigation of the perception-action circuitry much more feasible than in vertebrate species (Datta et al., 2008). While at the peripheral level exposure to the male emitted pheromone cis-vaccenyl acetate produces similar responses in males and females, the behavioral responses are completely different in the two sexes: females increase receptivity to courting males, whereas males show an increase of aggression towards males and suppression of courtship. These sexually dimorphic differences are mediated by anatomical and functional differences in the central nervous system. In particular, specific regions of the lateral horn receive projections from glomeruli linked to odor attraction and aversion, suggesting that valence is spatially encoded in this structure (Min et al., 2013). The link between lateral horn neurons and behavioral responses has not yet been entirely clarified but by tracing the pheromone-response circuit researchers have revealed lateral horn neurons with sexually dimorphic projections, including projections to male descending neurons, that enter the ventral nerve cord, which in turn controls movement (Ruta et al., 2010).

Unlearned preferences are widespread in the visual modality too. To make a few examples, fruit flies show phototactic responses (Gao et al., 2008; Yamaguchi et al., 2010), several insects have color preferences for flowers (Giurfa et al., 1995; Lunau and Maier, 1995; Gumbert, 2000) and naïve chicks display visual preferences for pecking objects (Hess and Gogel, 1954; Fantz, 1957) and for imprinting objects (Schaefer and Hess, 1951; Kovach, 1983, 1990; see also Rosa Salva et al., 2015 for a review about preferences for imprinting objects). The investigation of visual unlearned preferences in human infants has a long tradition: already in the Sixties Fantz (1963) showed a greater responsiveness to patterns than to hues. Recent work with children (8–16 years old) who gained sight after early-onset blindness, provides evidence of unlearned susceptibility to the Ponzo and Müller-Lyer illusions immediately after the onset of sight (Gandhi et al., 2015). This evidence points at the role of unlearned knowledge also in our species. We will discuss preferences for configurations of stimuli in human and non-human newborns in the next section. The complexity of the visual circuitry makes the investigation of the perception-action link more difficult than in the case of olfactory perception.

#### Structural Features and Structured Events

Beside sensory attraction and avoidance reactions for perceptual stimuli, predispositions to orient towards or avoid configurations of stimuli since the early stages of life have been identified in different species.

In the visual domain the case of reactions to face-like stimuli is paradigmatic. Stimuli that reproduce the configuration of faces, with two centered eye-like spots in the upper part and one centered mouth-like spot in the lower part, can trigger attraction responses. Few hours after birth, human newborns follow more effectively the movement of schematic face-like stimuli compared to scrambled faces, blank stimuli, upside-down face-like stimuli and top heavy stimuli (Goren et al., 1975; Johnson et al., 1991), and in the absence of visual experience with faces, naïve newly hatched chickens show an analog preference to approach face-like stimuli (Johnson and Horn, 1988; Rosa-Salva et al., 2010). This predisposition, shared between social species, has been attributed to a mechanism of preferential tuning towards care-givers, namely the CONSPEC mechanism (see Morton and Johnson, 1991). Eye-like stimuli can also elicit anti-predatory responses such as tonic immobility in chickens (Gallup and Nash, 1971; Gagliardi et al., 1976) and have been suggested to produce avoidance in insectivorous birds (Janzen et al., 2010; Hossie and Sherratt, 2013), although salience can be the major drive of this effect (Stevens et al., 2008). The neural circuits that mediate unlearned responses to faces and eye-like stimuli have not been completely identified, but some evidence suggests that in our species the initial attraction for faces might be mediated by subcortical areas (e.g., superior colliculus or homologous areas (Rosa Salva et al., 2015), while subsequent stages rely more on cortical areas (Johnson et al., 2014).

Precocial biases for biological *vs.* rigid motion (Vallortigara et al., 2005; Simion et al., 2008) and for self-propelled objects (Mascalzoni et al., 2010, 2013) can produce a preferential orientation towards structural and configurational patterns associated with animate entities. When points of light are attached to the joints of a moving animal kept in darkness, the animation conveys information about the animal’s activity that suggests the idea of biological motion Johansson (1973). Inexperienced newly hatched chicks (Vallortigara et al., 2005), as well as human newborns (Simion et al., 2008), show a spontaneous preference to approach/preferentially look at stimuli that move according to semi-rigid trajectories of a walking animal (biological motion) *vs*. the same animal rotating on the central axis in a rigid fashion (see also Rugani et al., 2015a). Naïve chicks (Mascalzoni et al., 2010) and human newborns (Di Giorgio et al., 2014) prefer to approach/fixate objects that exhibit self-produced motion compared to objects that move only after the contact with a moving object, and 5-month-old infants understand that objects can start to move only as a result of contact with another moving object, unless they are provided with inner energy that permits self-produced motion (Luo and Baillargeon, 2005).

In the acoustic modality, it has been shown that 1–3 days old newborn babies show differential hemispheric activation during the presentation of consonant *vs*. dissonant tones (Perani et al., 2010), suggesting early functional specialization for specific sounds. Moreover, 2 and 4-month old infants prefer to listen to consonant over dissonant intervals (Trainor et al., 2002). These preferences could be acquired not only through early exposure to music but also through embryonic experience, given the acoustic sensitivity of the fetus (for a review, see Hepper and Shahidullah, 1994). In the acoustic modality, Chiandetti and Vallortigara (2011a) have shown a precocial preference of newly hatched chicks for consonant *vs.* dissonant intervals, a phenomenon that could guide newly hatched chicks to attend to (and imprint on) animate rather than inanimate objects, given that the harmonic relationships between frequency components is associated with prominent features of sounds in natural environments.

These findings about unlearned preferences for structured stimuli that correlate with the presence of animate objects (face-like stimuli, biological motion, self-propelled objects, potentially consonant intervals) suggest that different vertebrate species may share a predisposed neural mechanism to detect animate objects (Vallortigara, 2012b). A core knowledge system for social cognition could have evolved to help newborns of social species in the interaction with their care-givers. Comparative work with precocial non-social species can help clarifying this issue.

### Predispositions for Learning and Unconditioned Assumptions

#### Predispositions for Preferential/Faster Learning of Specific Stimuli or Associations

Since the seminal work conducted on rats, which showed an easier association of audio-visual stimuli with electric shock and gustatory stimuli with gut sickness (Krechevsky, 1933; Garcia and Koelling, 1966), it is known that some predispositions exist that can ease or hinder the acquisition of specific associations. Other examples of species-specific biases, known as constraints on learning or preparedness for learning, include the preferential use of the “win-shift” over “win-stay” strategies in birds during foraging (Gill and Wolf, 1977; Kamil, 1978; Cole et al., 1982). The convenience of insects as model species has recently emerged in this field of research. Taking advantage of the learning abilities and fast life cycle of *D. melanogaster*, Dunlap and Stephens (2009, 2014) have used fruit flies to show the role of the environment in shaping learning mechanisms. By pairing an aversive taste contingency with color or with odor for dozens of subsequent generations, the authors found empirical support to the hypothesis that the reliability of associations encountered during the evolutionary history shapes the learning abilities of a species (Dunlap and Stephens, 2014). Experimental evolution on fruit flies has showed also the possibility to select for enhanced associative learning abilities in the olfactory domain (Mery and Kawecki, 2002). Organisms with a fast life cycle are extremely important to understand the evolutionary basis of cognitive mechanisms, including unlearned predispositions, at the behavioral and genetic level (Versace, 2015).

### Unconditioned Assumption/Expectations

Few months after birth, human babies understand that objects continue to exist even when they are no longer visible (object permanence, see Baillargeon et al., 1985), reason about the physical properties of objects such as height, size and solidity (Aguiar and Baillargeon, 1999, 2002), encode the location of objects (Newcombe et al., 1999) and encode causal relationships (Leslie and Keeble, 1987; Mascalzoni et al., 2013). This evidence though is compatible both with precocial learning and unlearned capabilities (or maturation of unlearned capabilities).

Increasing evidence collected with precocial species is clarifying to which extent unlearned knowledge about physical facts of the world can help animals to cope with the environment and in problem solving since the earliest stages of life. In spite of continuous changes in lightning, distance and point of view, different species have no difficulties in recognizing an object. Young chicks of nidifugal species develop an attachment and following response, known as filial imprinting (reviewed in Bateson, 1991), for the first conspicuous object encountered in their life. In nature this object is usually the mother hen. To understand whether chicks require experience with multiple appearances of the same object to produce invariant object recognition of the imprinting object, Wood and Wood (2015) have reared newly hatched chicks in a controlled imprinting environment with partial and impoverished experience with an imprinting object. Following exposure with only three views of a single object, chicks could build an invariant representation of it.

Object permanence has been investigated in young chicks that had no previous experience with occluded objects. By using imprinting objects disappearing behind occluders of different size, Chiandetti and Vallortigara (2011b) showed object permanence abilities in the absence of previous experience and spontaneous physical reasoning about occluded objects. In fact chicks expected that imprinting objects had disappeared more likely behind occluders bigger than their size than behind occluders smaller than their size.

Orientation, navigation and location memory rely on environmental cues such as the geometric features of the environment (Vallortigara et al., 2009). Newly hatched chicks are able to encode and use geometric information to search for a disappeared imprinting object in the absence of previous experience (Chiandetti et al., 2014). Fish (*Archocentrus nigrofasciatus*, Brown et al., 2007) as well as chicks of the domestic fowl (Chiandetti and Vallortigara, 2008) reared in home cages of different geometric shapes are equally capable of learning and performing navigational tasks using geometric information. These findings suggest that the effective use of geometric information does not require specific metric and geometrical experience (Chiandetti et al., 2014). Recent studies on bumble bees have shown that spontaneous use of geometrical features, boundaries and local environmental features (Lee and Vallortigara, 2015) can guide the untrained spatial behavior in these insects. Behavioral studies have just started to show the potential of insect models in the study of unlearned knowledge used in “high-level” cognitive abilities (Vallortigara et al., 1999).

Another striking example of precocious and untrained abilities is numerical cognition. Numerical abilities have been found in human infants (Xu et al., 2005; Libertus et al., 2009; Libertus and Brannon, 2010) and even newborns (Izard et al., 2009; Coubart et al., 2014; De Hevia et al., 2014). These studies offer good evidence for abstract numerical representations at the start of postnatal experience and parallel similar evidence obtained in newborn chicks (e.g., Rugani et al., 2014; and see also comments in Brugger, 2015; Drucker and Brannon, 2015). Infants habituated to pictures of the same number of objects (e.g., 8 dots) looked longer when presented with arrays of a new numerical value controlled for continuous variables (e.g., 16 dots; Xu, 2003; Brannon et al., 2004; Xu et al., 2005), thus showing an early sense of number/numerosity. Individual differences in this task remain stable during infancy (Libertus and Brannon, 2010; De Hevia and Spelke, 2010) and are a good predictor for subsequent mathematical abilities in childhood (Starr et al., 2013). Similarly to other domains, the learned/unlearned origin of this knowledge can be hardly established in human beings. Young chicks can be used to investigate precocial proto-mathematical cognition based on their need of proximity to social partners to reach an optimal temperature and avoid predators since the early stages of life. After imprinting, chicks treat imprinting objects as social partners and prefer to approach sets with larger number of imprinting objects, showing their untrained capability to distinguish between sets of different numerosities. This behavior does not simply reflect a general preference for larger sets or for larger extent of continuous features, since when presented with novel objects chicks approach the group with the same numerousness as in the original imprinting condition (Rugani et al., 2010) when continuous features are controlled. In chicks, even basic arithmetic skills are available at birth and in the absence of formal training, as shown by the capability to approach the larger set of imprinting objects that results after displacement (corresponding to addition or subtraction) of occluded imprinting objects (Rugani et al., 2009). In these experiments after imprinting the chicks saw a set of four and a set of one imprinting objects disappearing behind a screen. Subsequently chicks were shown imprinting objects moving between the screens. When left free to move, they chose to approach the screen that currently hide the largest number of objects, thus showing proto arithmetical capabilities to sum and subtract numerosities. Experiments run on numerical encoding in this species have clarified an untrained left-right representation of the ordinal value of numbers, suggesting that the left-right representation of the mental number line in human beings can reflect more a biological predisposition than mere cultural conditioning (Rugani et al., 2015b).

## Conclusions

In the absence of previous specific experience, unlearned knowledge can guide behavioral responses of approach and avoidance, and provide cues to problem solving. Establishing whether a response is guided by previous learning or it is rather spontaneously determined requires controlled rearing conditions until the moment of the experiment. By collecting evidence from disciplines as different as evolutionary biology, animal cognition and developmental psychology we have showed how different models and approaches can help clarifying the origins of precocious knowledge. When young individuals of altricial species are too immature to be tested before the acquisition of significant experience in the domain of interest, often precocial species are convenient models. This does not of course deny the existence of unlearned knowledge in human beings. There is indeed some straightforward evidence of this, as shown for example by some of the work with human newborns that provided evidence of abstract number at the start of postnatal life (see “Unconditioned Assumption/Expectations” Section). For instance, Izard et al. (2009) showed that newborn infants spontaneously associate stationary, visual-spatial arrays of 4–18 objects with auditory sequences of events on the basis of number. Here it seems unlikely (though perhaps it cannot be completely excluded) that newborns before the tests have had opportunities and time enough to associate, on the basis of their early minimal multimodal perceptions, visual and auditory stimuli which were time-locked and then to generalize the link to non time-locked stimulation. Besides, it is difficult to imagine how such a link can be formed without a predisposed abstract notion of number. Yet, in other cases it is virtually impossible to prevent human newborns from having even minimal visual experiences, and thus early learning, that can easily account for their precocious abilities (e.g., exposure to faces for predisposition to face-like stimuli, exposure to extended visual surfaces in the spatial layout for predisposition to geometry and so on).

Insect of species that do not require post-hatching parental care, such as the well-studied model *Drosophila melanogaster*, can be easily controlled until the moment of test and investigated at the behavioral, neurobiological and genetic level. Moreover, the relative simplicity of the olfactory circuits in insects makes the investigation of neural mechanisms feasible, and genetic analysis has already shed light into the genetic determinants of olfactory behavior (Rollmann et al., 2010; Arya et al., 2015). Other research has shown the potential of phylogenetically distant models for biomedical research on human beings (Reiter et al., 2001). Moreover, fruit flies have a life cycle fast enough to be monitored during experimental evolution for changes in their predispositions (and underlying genetic changes (Schlötterer et al., 2015; Versace, 2015). When selected for different associations (color/flavor *vs*. odor/flavor) fruit flies changed their promptness in learning the association used during selected (Dunlap and Stephens, 2014), thus indicating the evolutionary basis of preparedness for learning.

Avian precocial species, such chicks of the domestic fowl, have been extensively used to investigate unlearned knowledge about configurations of cues as well as unconditioned expectations about facts of the world that can to cope with the environment. The precocial preference for stimuli associated with the presence of animate objects that has been found in both naïve chicks and human newborns—e.g., face-like stimuli (Rosa-Salva et al., 2010), biological motion (Vallortigara et al., 2005; Simion et al., 2008)—suggests that species that require post-birth parental care might rely on an adaptive social core knowledge system moulded by natural selection. This hypothesis can be validated through comparative research that investigates the responses of precocial species with no post-hatching parental care, such as Chelonia. This example clearly shows that the interest in non-mammalian species is theoretically important, and not only justified by ethical reasons (e.g., primate infants cannot be controlled-reared until they reach the maturation required for behavioral tests), or by practical convenience (e.g., precocial avian species can be easily manipulated until the moment of the test and possess developed sensory and motor capabilities soon after hatching). The evidence collected in the last decades (Vallortigara, 2012b; reviewed in Spelke and Kinzler, 2007; Vallortigara et al., 2009; Spelke et al., 2010; Vallortigara, 2015), with the notable examples of unlearned number (e.g., Rugani et al., 2009, 2014) and spatial cognition (e.g., Vallortigara et al., 2009; Lee and Vallortigara, 2015) show the continuity of the human mind with other species, including phylogenetically distant species. By comparing different breeds and species it will possible to shed light into the adaptive value and evolutionary history of unlearned knowledge, and to understand the connection between sensory or “low-level” processes and other cognitive abilities, such as the link between the number sense in infancy and mathematical abilities in childhood (Starr et al., 2013). Furthermore, behavioral tests developed on precocial species, and therefore well-validated for their independence from specific experiential effects (again, social, space and number cognition provide excellent examples, see for instance (Haun et al., 2010; Vallortigara, 2012a,b) may set the stage for the development of similar tests in species that are not precocial but offer on the other hand peculiar advantages for molecular and genetic analyses (zebrafish is an obvious example, for social cognition see for instance Oliveira, 2013), for space cognition (see Lee et al., 2013; for number cognition see Potrich et al., 2015), thus favoring the step to translational research.

Precocial species have proven to have high translational value for areas associated with neurocognitive disturbances and developmental disorders. Van der Voet et al. (2014) have recently reviewed the importance of *Drosophila* for translational research on dozens of early onset cognitive disorders, from Fragile X syndrome (the most extensively studied) to Autism spectrum disorders (e.g., Hahn et al., 2013 for impairment of social behaviors in mutant flies), highlighting the convenience of fruit flies to investigate gene function, determine primary origin of pathology and identifying specific genes involved, test hypotheses on *in vivo* models, acquire disease-relevant tissues, combine mutations in different genes to dissect molecular networks. Genetic and neurobiological approaches can be conveniently applied to fruit flies to study unlearned behaviors and clarify the role of environmental cues in eliciting specific responses, circuits and computations involved in pre-programmed behaviors (Manoli et al., 2006). Vertebrate non-mammalian species are also highly valuable translational models. For instance, besides being a classical model for epilepsy (see for instance Douaud et al., 2011 for recent studies) the domestic chick is a well-established model for the study of the cellular mechanisms of memory and potential therapies for dementia diseases (Mileusnic and Rose, 2010). Furthermore, studies on precocial social species have permitted the formulation of original hypothesis on some developmental disturbances such as autism. The so-called “social orienting hypothesis” assumes that unusual development of the social brain in individuals with autism spectrum disorders may be due to an alteration in the early activation of a subcortical mechanism that biases newborns to orient to relevant social visual stimuli (Johnson, 2005). The hypothesis was originally formulated in the context of studies on chicks spontaneous preferences (Morton and Johnson, 1991; Rosa Salva et al., 2011) and recent work with this animal model inspired recent attempts to develop early bio-markers of these neurodevelopmental disorders (Di Giorgio et al., 2014; Johnson, 2014; Rosa Salva et al., 2015) and the study of the underlying molecular mechanisms (Nishigori et al., 2013).

## Conflict of Interest Statement

The authors declare that the research was conducted in the absence of any commercial or financial relationships that could be construed as a potential conflict of interest.

## Footnotes

^1^Similar limitations apply to other altricial species, as certain cichlid fish, that require an extended period of development before reaching a complete sensory-motor maturation and behavioral performance (Barlow, 2000). Fish species such as the zebrafish (*Danio rerio*), showed to be convenient models for the investigation of social cognition (Oliveira, 2013).

^2^This approach can clarify which input is not necessary for the development of a certain behavior, and not that no experience is not required for the development of a certain behavior, because a certain amount of experience is always present.
